# Association of chemotherapy and stoma features with peristomal skin disorders in rectal cancer patients

**DOI:** 10.1038/s41598-025-26387-1

**Published:** 2025-11-26

**Authors:** Takuya Shiraishi, Yuji Nishizawa, Mifumi Nakajima, Ryoko Kado, Hiroomi Ogawa, Satoh Naomi, Yohei Owada, Tsuyoshi Enomoto, Shinji Yazawa, Yukihiro Hamahata, Yumi Isogami, Kazuo Kitagawa, Maki Sakamoto, Hiroya Enomoto, Akiko Egawa, Daichi Kitaguchi, Hiro Hasegawa, Koji Ikeda, Yuichiro Tsukada, Masaaki Ito

**Affiliations:** 1 Department of General Surgical Science, Gunma University Graduate School of Medicine, Maebashi, Japan; 2 Department of Colorectal Surgery, National Cancer Center Hospital East, Kashiwa, Japan; 3 Department of Nursing, National Cancer Center Hospital East, Kashiwa, Japan; 4 Department of Nursing, Gunma University Hospital, Maebashi, Japan; 5 Department of Gastrointestinal and Hepatobiliary Pancreatic Surgery, University of Tsukuba, Tsukuba, Japan; 6 Department of Nursing, University of Tsukuba Hospital, Tsukuba, Japan; 7 Department of Colorectal Surgery, Tsujinaka Hospital Kashiwanoha, Kashiwa, Japan; 8 Department of Nursing, Tsujinaka Hospital Kashiwanoha, Kashiwa, Japan; 9 Department of Surgery, The Jikei University Kashiwa Hospital, Kashiwa, Japan; 10 Department of Nursing, The Jikei University Kashiwa Hospital, Kashiwa, Japan; 11 Department of Surgery, The Jikei University Daisan Hospital, Komae, Japan; 12 Department of Nursing, The Jikei University Hospital, Tokyo, Japan

**Keywords:** Chemotherapy-induced complications, Colorectal neoplasms, Diabetes mellitus, Peristomal skin complications, Stoma care, Risk factors, Outcomes research, Surgical oncology, Disease prevention

## Abstract

This study aimed to identify factors associated with the onset and worsening of peristomal skin disorders during outpatient follow-up after stoma creation for malignant rectal tumors. This prospective multicenter observational study enrolled patients who underwent stoma creation for malignant rectal tumors between December 2019 and December 2021 at six Japanese institutions. Patients were evaluated using the ABCD-Stoma and DET scoring systems at 1 month and during subsequent follow-up visits. A total of 130 patients were analyzed. At the 1-month visit, 53 (40.8%) had no peristomal skin disorders and 77 (59.2%) had lesions. Multivariate analysis showed that chemotherapy (odds ratio [OR] = 17.50, 95% confidence interval [CI]: 1.76–174.27, *p* = 0.015), loop stoma (OR = 43.46, 95%CI 1.70–1113.99, *p* = 0.023), and stoma height < 10 mm (OR = 32.68, 95%CI: 1.94–549.55, *p* = 0.015) were independently associated with the development of peristomal skin disorders. Diabetes mellitus (OR = 4.26, 95%CI: 1.08–16.79, *p* = 0.039) was independently associated with worsening, whereas stoma height < 10 mm (OR = 5.85, 95%CI: 1.16–29.35, *p* = 0.032) was associated with severe lesions during follow-up. These findings highlight the importance of continuous outpatient monitoring and tailored stoma-care support to facilitate early detection and management of peristomal skin disorders, especially in high-risk patients.

## Introduction

In rectal cancer surgery, a temporary stoma is often created to prevent severe complications from anastomotic leakage, whereas a permanent stoma is constructed when anus preservation is not feasible. Stoma-related complications occur in over 80% of cases, leading to a reduced quality of life (QOL) and increased medical costs, highlighting the need for appropriate ongoing care^1–3^. With advancements in medical technology and perioperative management, postoperative hospital stays have substantially shortened, underscoring the growing importance of stoma outpatient visits to provide continued support after discharge.

Among stoma-related complications, peristomal skin disorders are the most common, affecting up to 80% of patients, and are considered a frequent issue for individuals with a stoma^4^. These skin complications cause itching and pain, considerably impairing the QOL, and increase the frequency and cost of appliance replacement^3,5,6^. Because peristomal skin disorders may progress rapidly if not recognized early, identifying patients at high risk of onset or worsening during outpatient follow-up is clinically important. However, patients often fail to recognize these skin changes, with fewer than half being aware of their condition^7,8^. Clarifying the associated risk factors can facilitate timely intervention and improve the quality of stoma care.

Several standardized tools have been developed to objectively assess peristomal skin conditions. These include the ABCD-Stoma^®^ score and the Discoloration, Erosion, Tissue overgrowth (DET) score, which quantify lesion severity and monitor changes over time^9,10^. These instruments enable reproducible assessment and are widely applied in clinical research and practice.

Although previous studies have reported various potential risk factors, including chemotherapy, stoma type, and height^11–13^, no prospective study has identified factors associated with the onset or worsening of these conditions during outpatient follow-up. Therefore, this study aimed to identify factors associated with the onset and worsening of peristomal skin disorders during outpatient follow-up after stoma creation for malignant rectal tumors. Particular attention was given to chemotherapy exposure, stoma type, and stoma height.

## Methods

This was a prospective, multicenter observational cohort study conducted at six high-volume regional hospitals in Japan. To identify factors associated with the onset and worsening of peristomal skin disorders, the study used the ABCD-Stoma system as a standardized assessment tool^14^.

### Enrolment

Patients with malignant rectal tumors, regardless of stage, who were scheduled for surgical procedures involving stoma formation at the participating institutions, were considered eligible for inclusion. The enrollment period spanned from December 2019 to December 2021.

The study included patients who met the following criteria: (1) a diagnosis of malignant rectal tumors, such as adenocarcinoma, confirmed through histological analysis; (2) the presence of either a temporary or permanent stoma; (3) any clinical stage of the disease; (4) primary or recurrent tumors; (5) aged 20 years or older at the time of registration; (6) initiation of chemotherapy confirmed after the initial evaluation during the follow-up period; and (7) provision of informed consent for the collection of observational data. Patients were excluded from the study if any of the following applied: (1) chemoradiotherapy was performed before or after surgery, because radiation-induced dermatitis could confound peristomal findings; (2) essential data such as ABCD-Stoma or DET scores were missing (“insufficient data”); (3) scheduled outpatient assessments could not be conducted according to protocol; or (4) the patient was lost to follow-up within 3 months or otherwise judged unsuitable by the investigators.

The research was conducted in full compliance with the ethical principles outlined in the Declaration of Helsinki. The finalized study protocol, along with the participant information sheet and consent forms, was formally approved by the Ethics Committee of the National Cancer Center East Hospital (Approval No. 2019 − 204) and the Institutional Review Boards at all collaborating institutions. Written informed consent was obtained from all participants prior to their enrollment in the study.

### Definitions and criteria for the worsening of peristomal skin disorders

All assessments were performed by experienced Wound, Ostomy, and Continence Nurses and colorectal surgeons. For the consistency, a standardized evaluation manual was distributed to all six participating institutions. Furthermore, prior to study initiation, assessors were provided with an opportunity to review and align their understanding of the evaluation methods for both the ABCD-Stoma and DET scoring systems. These measures ensured that the evaluation criteria and scoring thresholds were standardized across institutions.

Peristomal skin disorders were evaluated based on skin lesions, including erythema, erosion, blisters, pustules, ulcers, tissue overgrowth, and pigmentation. The severity of these conditions was assessed using the ABCD-Stoma and Discoloration, Erosion, and Tissue overgrowth (DET) scoring systems^9,10^.

The ABCD-Stoma score, ranging from 0 to 45, classifies normal skin at a score of 0 and the most severe peristomal skin disorder at 45. A score of ≥ 4 on the ABCD-Stoma scale indicates severe peristomal skin disorders, correlating with a treatment period exceeding 28 days^9^. The DET score evaluates discoloration, erosion, and tissue overgrowth, ranging from 0 to 15, where 0 indicates no skin issues and 15 represents the most severe and extensive skin conditions. A DET score of ≥ 7 is considered indicative of severe peristomal skin disorders^10,15^.

Worsening of peristomal skin disorders was defined as an increase of one or more points in either the ABCD-Stoma score or DET score compared with the previous evaluation.

### Follow-up schedule

The postoperative inpatient observation and examination schedule adhered to the standard protocols established at each participating facility. The assessment of peristomal skin disorders, being a critical aspect of the analysis, was conducted with meticulous care following the strict criteria outlined in the “Definition of Peristomal Skin Disorders.”

For post-discharge observation and examinations, patients were monitored at intervals of 1, 3, 6, 9, and 12 months after surgery (within 2 weeks before or after each designated time point) in accordance with the standard outpatient schedules of each facility. Thus, the observation period extended up to 12 months post-surgery. The monitoring period was considered complete if stoma closure was performed during this observation period. During routine stoma follow-up, interventions to address stoma-related complications were implemented. In such cases, ABCD-Stoma Care was primarily utilized as a guide for establishing appropriate skin care goals and methods^14^.

### Statistical analysis

Continuous variables are presented as medians and range, and categorical variables as numbers and percentages. Group comparisons were performed using the Mann–Whitney U test for continuous variables and Fisher’s exact test for categorical variables. Missing data were handled by listwise deletion.

At the outpatient visit 1 month after surgery, patients were classified into two groups: without peristomal skin disorders and with peristomal skin disorders. To identify risk factors associated with the development of peristomal skin disorders, worsening of peristomal skin disorders, and the presence of severe peristomal skin disorders during outpatient follow-up after 1 month, variables with *p* < 0.05 in univariate analyses were entered into multivariate logistic regression using a forced-entry method.

All statistical analyses were conducted using SPSS (version 29.0; IBM Corp., Armonk, NY), with the threshold for statistical significance set at *p* < 0.05.

## Results

### Patient inclusion and exclusion

**Fig. 1:**
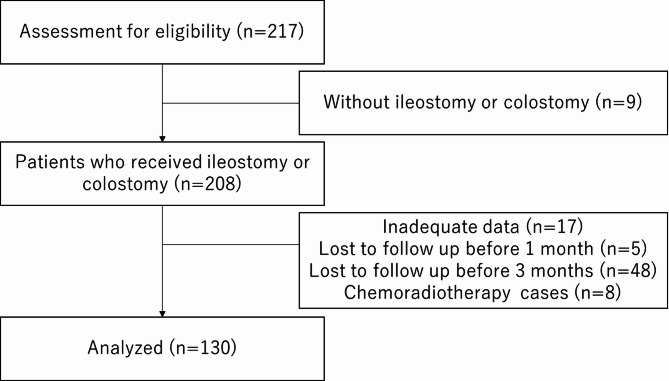
Diagram of patient selection for the study.

This prospective, multicenter observational study screened 217 patients between December 2019 and December 2021 to evaluate their eligibility. After excluding 87 patients—9 without ileostomy or colostomy, 17 with missing ABCD-Stoma or DET data (“insufficient data”), 5 lost to follow-up within 1 month, 48 lost to follow-up within 3 months, and 8 who received chemoradiotherapy before or after surgery—a total of 130 patients were included in the final analysis (Fig. 1).

### Patient characteristics

Table 1 presents the patient characteristics. Of the 130 patients, 96 (73.8%) were male and 34 (26.2%) female. The median age was 65 years (range: 23–86 years) and median body mass index (BMI) 22.9 kg/m² (range: 13.9–32.2 kg/m²). No patient underwent emergency surgery for stoma creation. Additionally, all stoma sites were preoperatively marked by Wound, Ostomy, and Continence Nurses. Colostomies were performed in 37 patients (28.5%), while ileostomies were created in 93 (71.5%). End stomas were observed in 29 patients (22.3%), whereas loop stomas were present in 101 (77.7%). The median stoma height was 14 mm (range: 0–50 mm). Regarding postoperative treatment, 61 patients (46.9%) underwent surgery alone, while 69 (53.1%) received chemotherapy.

**Table 1:** Patient characteristics.

Sex, *N* (%)	Male	96 (73.8)
	Female	34 (26.2)
Age (years), median (range)		65 (23–86)
BMI (kg/m^2^), median (range)		22.9 (13.9–32.2)
Smoker, N (%)	No current	100 (76.9)
	Current	30 (23.1)
DM, N (%)		19 (14.6)
Hypertension, N (%)		43 (33.1)
Cardiovascular disease, N (%)		9 (6.9)
Preoperative treatments, N (%)		40 (30.8)
Hb (g/dL), median (range)		13.4 (3.8–18.1)
Alb (g/dL), median (range)		4.1 (2.6–5.1)
Cr (mg/dL), median (range)		0.78 (0.47–3.38)
GOT (IU/L), median (range)		20 (9–53)
GPT (IU/L), median (range)		17 (6–71)
Approach type, N (%)	Open	4 (3.1)
	Lap or Rob	126 (96.9)
Stoma type-1, N (%)	Colostomy	37 (28.5)
	Ileostomy	93 (71.5)
Stoma type-2, N (%)	End	29 (22.3)
	Loop	101 (77.7)
Using the skin bridge method, N (%)		73 (56.2)
Height of stoma (mm), median (range)		14 (0–50)
Base area of stoma (mm^2^), median (range)		10.2 (4.40–24.44)
Oblateness of stoma (mm^2^), median (range)		0.10 (0.000–0.455)
Peristomal skin disorders at 1 month evaluated by ABCD-Stoma, N (%)	No	53 (40.8)
	Yes	77 (59.2)
Severe peristomal skin disorders evaluated by ABCD-Stoma, N (%)		7 (5.4)
Peristomal skin disorders at 1 month evaluated by DET score, N (%)	No	53 (40.8)
	Yes	77 (59.2)
Severe peristomal skin disorders evaluated by DET score, N (%)		0 (0.0)
Chemotherapy, N (%)	No	61 (46.9)
	Yes	69 (53.1)

BMI, Body Mass Index; DM, Diabetes Mellitus; Hb, Hemoglobin; Alb, Albumin; Cr, Creatinine; GOT, Glutamate Oxaloacetate Transaminase; GPT, Glutamate Pyruvate Transaminase; Lap, Laparoscopic; Rob, Robotic; ABCD-Stoma, Arousal, Body, Color, and Depth Stoma Classification; DET score, Discoloration, Erosion, and Tissue Overgrowth Score.

### Peristomal skin disorders at 1-month and during follow-up

At the 1-month outpatient visit, peristomal skin disorders were detected in 77 patients (59.2%) and absent in 53 (40.8%) according to ABCD-Stoma scale. Similarly, evaluation using the DET score showed identical results, with 77 patients (59.2%) having peristomal skin disorders and 53 (40.8%) unaffected at the 1-month outpatient visit.

Following outpatient follow-up, severe peristomal skin disorders (ABCD-Stoma score ≥ 4) developed in seven patients (5.4%), all of whom had already exhibited the condition at the 1-month visit. In contrast, no cases of severe peristomal skin disorders (DET score ≥ 7) were observed after outpatient follow-up.

### Risk factors for peristomal skin disorders in patients without peristomal skin disorders at 1-month follow-up

Table 2 presents the univariate and multivariate analyses of risk factors for peristomal skin disorders during outpatient follow-up in patients who had no peristomal skin disorders at the 1-month outpatient visit, as assessed using ABCD-Stoma or the DET score. Univariate analysis identified three significant risk factors: the presence of a loop stoma (*p* = 0.028), a stoma height of < 10 mm (*p* = 0.027), and undergoing chemotherapy (*p* = 0.019), all of which were associated with the development of peristomal skin disorders during follow-up. In contrast, other variables—including sex, age, BMI, smoking status, diabetes mellitus (DM), hypertension, cardiovascular disease, preoperative treatments, hemoglobin, albumin, creatinine (Cr), glutamic oxaloacetic transaminase (GOT), glutamic pyruvic transaminase (GPT), approach type, stoma type (colostomy or ileostomy), use of the skin bridge method, base area of stoma, oblateness of the stoma, and history of peristomal skin disorders—were not significantly associated with the development of peristomal skin disorders during follow-up (all *p* > 0.05). Multivariate analysis showed that these variables were independently associated with the development of peristomal skin disorders, with loop stoma (odds ratio [OR], 43.463; 95% confidence interval [CI]: 1.696–1113.992; *p* = 0.023), stoma height < 10 mm (OR, 32.679; 95% CI: 1.943–549.550; *p* = 0.015), and chemotherapy (OR, 17.503; 95% CI: 1.758–174.265; *p* = 0.015) showing significant associations with peristomal skin disorder development.

**Table 2:** Risk factors for peristomal skin disorders in patients without peristomal skin disorders at 1-month outpatient visit.

		No PSD, *N* = 40	PSD, *N* = 13	P-value	Multivariate	
					OR (95% CI)	P-value
Sex, N (%)	Male	25 (62.5)	10 (76.9)	0.274		
	Female	15 (37.5)	3 (23.2)			
Age (years), median (range)		56.5 (34.0–85.0)	62.0 (39.0–74.0)	0.934		
BMI (kg/m^2^), median (range)		23.5 (13.9–32.2)	23.6 (16.6–31.6)	0.836		
Smoker, N (%)	No current	32 (80.0)	8 (61.5)	0.165		
	Current	8 (20.0)	5 (38.5)			
DM, N (%)		3 (7.5)	2 (15.4)	0.357		
Hypertension, N (%)		11 (27.5)	3 (23.1)	0.531		
Cardiovascular disease, N (%)		1 (2.5)	0 (0.0)	0.755		
Preoperative treatments, N (%)		14 (35.0)	5 (38.5)	0.536		
Hb (g/dL), N (%)	≤ 10	4 (10.0)	1 (7.7)	0.643		
	> 10	36 (90.0)	12 (92.3)			
Alb (g/dL), N (%)	≤ 3.5	9 (2.5)	3 (23.1)	0.619		
	> 3.5	31 (77.5)	10 (76.9)			
Cr (mg/dL), N (%)	≤ 1.0	33 (82.5)	12 (92.3)	0.360		
	> 1.0	7 (17.5)	1 (7.7)			
GOT (IU/L), N (%)	≤ 30	37 (92.5)	12 (92.3)	0.688		
	> 30	3 (7.5)	1 (7.7)			
GPT (IU/L), N (%)	≤ 30	35 (87.5)	12 (92.3)	0.540		
	> 30	5 (12.5)	1 (7.7)			
Approach type, N (%)	Open	3 (7.5)	0 (0.0)	0.422		
	Lap or Rob	37 (92.5)	13 (100.0)			
Stoma type-1, N (%)	Colostomy	18 (45.0)	6 (46.2)	0.942		
	Ileostomy	22 (55.0)	7 (53.8)			
Stoma type-2, N (%)	End	16 (40.0)	1 (7.7)	0.028	Reference	0.023
	Loop	24 (60.0)	12 (92.3)		43.463 (1.696–1113.992)	
Using the skin bridge method, N (%)		18 (45.0)	7 (53.8)	0.579		
Height of stoma (mm), N (%)	≥ 10	38 (95.0)	9 (69.2)	0.027	Reference	0.015
	< 10	2 (5.0)	4 (30.8)		32.679 (1.943–549.550)	
Base area of stoma (mm^2^), median (range)		10.60 (5.75–22.50)	11.20 (7.83–16.50)	0.482		
Oblateness of stoma (mm^2^), median (range)		0.080 (0.000–0.313)	0.069 (0.000–0.455)	0.917		
History of peristomal skin disorders, N (%)	No	31 (77.5)	8 (61.5)	0.217		
	Yes	9 (22.5)	5 (38.5)			
Chemotherapy, N (%)	No	21 (52.5)	2 (15.4)	0.019	Reference	0.015
	Yes	19 (47.5)	11 (84.6)		17.503 (1.758–174.265)	

PSD, Peristomal Skin Disorder; OR, Odds Ratio; CI, Confidence Interval; BMI, Body Mass Index; DM, Diabetes Mellitus; Hb, Hemoglobin; Alb, Albumin; Cr, Creatinine; GOT, Glutamate Oxaloacetate Transaminase; GPT, Glutamate Pyruvate Transaminase; Lap, Laparoscopic; Rob, Robotic.

### Risk factors for worsening peristomal skin disorders in patients with peristomal skin disorders at 1-month follow-up (ABCD-Stoma)

Table 3 presents the univariate and multivariate analyses of risk factors for the worsening of peristomal skin disorders during outpatient follow-up in patients who had peristomal skin disorders at the 1-month outpatient visit, as assessed by ABCD-Stoma. Univariate analysis identified three significant risk factors: DM (*p* = 0.024), serum albumin ≤ 3.5 g/dL (*p* = 0.042), and oblateness of the stoma (*p* = 0.039), all of which were associated with worsening peristomal skin disorders. In contrast, other variables—including sex, age, BMI, smoking status, hypertension, cardiovascular disease, preoperative treatments, hemoglobin, Cr, GOT, GPT, approach type, stoma type (colostomy or ileostomy), stoma type (end or loop), use of the skin bridge method, height of stoma, base area of stoma, and chemotherapy—were not significantly associated with worsening peristomal skin disorders (all *p* > 0.05). Multivariate analysis showed that DM (OR, 4.258; 95% CI: 1.080–16.791; *p* = 0.039) was independently associated with the worsening of peristomal skin disorders.

**Table 3:** Risk factors for the worsening of peristomal skin disorders in patients with peristomal skin disorders at 1-month outpatient visit evaluated by ABCD-Stoma.

		No worsening, *N* = 62	Worsening, *N* = 15	P-value	Multivariate	
					OR (95% CI)	P-value
Sex, N (%)	Male	49 (79.0)	12 (80.0)	0.622		
	Female	13 (21.0)	3 (20.0)			
Age (years), median (range)		65.0 (23.0–86.0)	72.0 (48.0–84.0)	0.054		
BMI (kg/m^2^), median (range)		22.9 (14.5–29.2)	21.4 (17.9–31.3)	0.181		
Smoker, N (%)	No current	50 (80.6)	10 (66.7)	0.201		
	Current	12 (19.4)	5 (33.3)			
DM, N (%)		8 (12.9)	6 (40.0)	0.024	4.258 (1.080–16.791)	0.039
Hypertension, N (%)		24 (38.7)	5 (33.3)	0.700		
Cardiovascular disease, N (%)		6 (9.7)	2 (13.3)	0.489		
Preoperative treatments, N (%)		19 (30.6)	2 (13.3)	0.152		
Hb (g/dL), N (%)	≤ 10	4 (6.5)	3 (20.0)	0.130		
	> 10	58 (93.5)	12 (80.0)			
Alb (g/dL), N (%)	≤ 3.5	4 (6.5)	4 (26.7)	0.042	3.099 (0.571–16.827)	0.190
	> 3.5	58 (93.5)	11 (73.3)			
Cr (mg/dL), N (%)	≤ 1.0	56 (90.3)	13 (86.7)	0.489		
	> 1.0	6 (9.7)	2 (13.3)			
GOT (IU/L), N (%)	≤ 30	54 (87.1)	14 (93.3)	0.440		
	> 30	8 (12.9)	1 (6.7)			
GPT (IU/L), N (%)	≤ 30	49 (79.0)	12 (80.0)	0.622		
	> 30	13 (21.0)	3 (20.0)			
Approach type, N (%)	Open	1 (1.6)	0 (0.0)	0.805		
	Lap or Rob	61 (98.4)	15 (100.0)			
Stoma type-1, N (%)	Colostomy	9 (14.5)	4 (26.7)	0.222		
	Ileostomy	53 (85.5)	11 (73.3)			
Stoma type-2, N (%)	End	9 (14.5)	3 (20.0)	0.427		
	Loop	53 (85.5)	12 (80.0)			
Using the skin bridge method, N (%)		37 (59.7)	11 (73.3)	0.327		
Height of stoma (mm), N (%)	≥ 10	51 (82.3)	9 (60.0)	0.069		
	< 10	11 (17.7)	6 (40.0)			
Base area of stoma (mm^2^), median (range)		9.60 (4.40–22.50)	8.99 (6.00–24.44)	0.524		
Oblateness of stoma (mm^2^), median (range)		0.118 (0.000–0.429)	0.069 (0.000–0.390)	0.039	0.005 (0.000–7.981)	0.159
Chemotherapy, N (%)	No	31 (50.0)	7 (46.7)	0.817		
	Yes	31 (50.0)	8 (53.3)			

PSD, Peristomal Skin Disorder; OR, Odds Ratio; CI, Confidence Interval; BMI, Body Mass Index; DM, Diabetes Mellitus; Hb, Hemoglobin; Alb, Albumin; Cr, Creatinine; GOT, Glutamate Oxaloacetate Transaminase; GPT, Glutamate Pyruvate Transaminase; Lap, Laparoscopic; Rob, Robotic.

### Risk factors for worsening peristomal skin disorders in patients with peristomal skin disorders at 1-month follow-up (DET score)

Table 4 displays the univariate and multivariate analyses of the risk factors for the worsening of peristomal skin disorders during outpatient follow-up in patients with peristomal skin disorders at the 1-month outpatient visit, as evaluated by the DET score. Both univariate and multivariate analyses revealed that no significant risk factors were identified for the worsening of peristomal skin disorders during outpatient follow-up in these patients when evaluated by the DET score.

**Table 4:** Risk factors for the worsening of peristomal skin disorders in patients with peristomal skin disorders at 1-month outpatient visit evaluated by DET score.

		No worsening, *N* = 61	Worsening, *N* = 16	P-value	Multivariate	
					OR (95% CI)	P-value
Sex, N (%)	Male	49 (80.3)	12 (75.0)	0.436		
	Female	12 (19.7)	4 (25.0)			
Age (years), median (range)		65.0 (33.0–86.0)	67.5 (23.0–84.0)	0.841		
BMI (kg/m^2^), median (range)		22.9 (18.1–31.3)	21.1 (14.5–29.1)	0.075		
Smoker, N (%)	No current	46 (75.4)	14 (87.5)	0.250		
	Current	15 (24.6)	2 (12.5)			
DM, N (%)		11 (18.0)	3 (18.8)	0.599		
Hypertension, N (%)		25 (41.0)	4 (25.0)	0.240		
Cardiovascular disease, N (%)		7 (11.5)	1 (6.3)	0.472		
Preoperative treatments, N (%)		19 (31.1)	2 (12.5)	0.117		
Hb (g/dL), N (%)	≤ 10	4 (6.6)	3 (18.8)	0.152		
	> 10	57 (93.4)	13 (81.3)			
Alb (g/dL), N (%)	≤ 3.5	5 (8.2)	3 (18.8)	0.212		
	> 3.5	56 (91.8)	13 (81.3)			
Cr (mg/dL), N (%)	≤ 1.0	55 (90.2)	14 (87.5)	0.528		
	> 1.0	6 (9.8)	2 (12.5)			
GOT (IU/L), N (%)	≤ 30	56 (91.8)	12 (75.0)	0.083		
	> 30	5 (8.2)	4 (25.0)			
GPT (IU/L), N (%)	≤ 30	48 (78.7)	13 (81.3)	0.564		
	> 30	13 (21.3)	3 (18.8)			
Approach type, N (%)	Open	1 (1.6)	0 (0.0)	0.792		
	Lap or Rob	60 (98.4)	16 (100.0)			
Stoma type-1, N (%)	Colostomy	10 (16.4)	3 (18.8)	0.540		
	Ileostomy	51 (83.6)	13 (81.3)			
Stoma type-2, N (%)	End	9 (14.8)	3 (18.8)	0.476		
	Loop	52 (85.2)	13 (81.3)			
Using the skin bridge method, N (%)		38 (62.3)	10 (62.5)	0.988		
Height of stoma (mm), N (%)	≥ 10	48 (78.7)	12 (75.0)	0.493		
	< 10	13 (21.3)	4 (25.0)			
Base area of stoma (mm^2^), median (range)		9.60 (5.00–24.44)	8.11 (4.40–18.40)	0.138		
Oblateness of stoma (mm^2^), median (range)		0.108 (0.000–0.429)	0.095 (0.000–0.343)	0.611		
Chemotherapy, N (%)	No	29 (47.5)	9 (56.3)	0.535		
	Yes	32 (52.5)	7 (43.8)			

DET, Discoloration, Erosion, and Tissue Overgrowth; PSD, Peristomal Skin Disorder; OR, Odds Ratio; CI, Confidence Interval; BMI, Body Mass Index; DM, Diabetes Mellitus; Hb, Hemoglobin; Alb, Albumin; Cr, Creatinine; GOT, Glutamate Oxaloacetate Transaminase (Aspartate Aminotransferase, AST); GPT, Glutamate Pyruvate Transaminase (Alanine Aminotransferase, ALT); Lap, Laparoscopic; Rob, Robotic.

### Risk factors for severe peristomal skin disorder development in patients with peristomal skin disorders at 1-month follow-up (ABCD-Stoma)

Table 5 presents the univariate and multivariate analyses of the risk factors for the development of severe peristomal skin disorders during outpatient follow-up in patients with peristomal skin disorders at the 1-month outpatient visit, as evaluated by the ABCD-Stoma. Univariate analysis revealed that a stoma height of < 10 mm (*p* = 0.039) was significantly associated with the development of severe peristomal skin disorders during outpatient follow-up. In contrast, other variables—including sex, age, BMI, smoking status, DM, hypertension, cardiovascular disease, preoperative treatments, hemoglobin, albumin, Cr, GOT, GPT, approach type, stoma type (colostomy or ileostomy), stoma type (end or loop), use of the skin bridge method, base area of stoma, oblateness of stoma, and chemotherapy—were not significantly associated with the development of severe peristomal skin disorders during follow-up (all *p* > 0.05). Multivariate analysis showed that a stoma height of < 10 mm (OR, 5.846; 95% CI: 1.164–29.353; *p* = 0.032) was independently associated with the development of severe peristomal skin disorders during outpatient follow-up in these patients.

**Table 5:** Risk factors for the development of severe peristomal skin disorders in patients with peristomal skin disorders at 1-month outpatient visit evaluated by ABCD-Stoma.

		No severe PSD, *N* = 70	Severe PSD, *N* = 7	P-value	Multivariate	
					OR (95% CI)	P-value
Sex, N (%)	Male	55 (78.6)	6 (85.7)	0.551		
	Female	15 (21.4)	1 (14.3)			
Age (years), median (range)		65.5 (23.0–86.0)	70.0 (60.0–84.0)	0.136		
BMI (kg/m^2^), median (range)		22.5 (14.5–29.2)	22.8 (19.2–21.3)	0.547		
Smoker, N (%)	No current	56 (80.0)	4 (57.1)	0.177		
	Current	14 (20.0)	3 (42.9)			
DM, N (%)		11 (15.7)	3 (42.9)	0.108		
Hypertension, N (%)		26 (37.1)	3 (42.9)	0.532		
Cardiovascular disease, N (%)		7 (10.0)	1 (14.3)	0.551		
Preoperative treatments, N (%)		19 (27.1)	2 (28.6)	0.620		
Hb (g/dL), N (%)	≤ 10	6 (8.6)	1 (14.3)	0.502		
	> 10	64 (91.4)	6 (85.7)			
Alb (g/dL), N (%)	≤ 3.5	6 (8.6)	2 (28.6)	0.153		
	> 3.5	64 (91.4)	5 (71.4)			
Cr (mg/dL), N (%)	≤ 1.0	63 (90.0)	6 (85.7)	0.551		
	> 1.0	7 (10.0)	1 (14.3)			
GOT (IU/L), N (%)	≤ 30	61 (87.1)	7 (100.0)	0.403		
	> 30	9 (12.9)	0 (0.0)			
GPT (IU/L), N (%)	≤ 30	55 (78.6)	6 (85.7)	0.551		
	> 30	15 (21.4)	1 (14.3)			
Approach type, N (%)	Open	1 (1.4)	0 (0.0)	0.909		
	Lap or Rob	69 (98.6)	7 (100.0)			
Stoma type-1, N (%)	Colostomy	12 (7.1)	1 (14.3)	0.664		
	Ileostomy	58 (82.9)	6 (85.7)			
Stoma type-2, N (%)	End	11 (15.7)	1 (14.3)	0.702		
	Loop	59 (84.3)	6 (85.7)			
Using the skin bridge method, N (%)		43 (61.4)	5 (71.4)	0.468		
Height of stoma (mm), N (%)	≥ 10	57 (81.4)	3 (42.9)	0.039	Reference	0.032
	< 10	13 (18.6)	4 (57.1)		5.846 (1.164–29.353)	
Base area of stoma (mm^2^), median (range)		9.60 (4.40–24.44)	8.10 (6.00–11.55)	0.288		
Oblateness of stoma (mm^2^), median (range)		0.111 (0.000–0.429)	0.069 (0.040–0.390)	0.343		
Chemotherapy, N (%)	No	33 (47.1)	5 (71.4)	0.205		
	Yes	37 (52.9)	2 (28.6)			

PSD, Peristomal Skin Disorder; OR, Odds Ratio; CI, Confidence Interval; BMI, Body Mass Index; DM, Diabetes Mellitus; Hb, Hemoglobin; Alb, Albumin; Cr, Creatinine; GOT, Glutamate Oxaloacetate Transaminase; GPT, Glutamate Pyruvate Transaminase; Lap, Laparoscopic; Rob, Robotic.

## Discussion

Among patients who did not have peristomal skin disorders at the 1-month outpatient visit, the introduction of chemotherapy was independently associated with the development of peristomal skin disorders during outpatient follow-up. A previous retrospective study has also reported an association between adjuvant chemotherapy and peristomal skin disorders^16^. The mechanisms underlying this relationship may include chemotherapy-induced cytotoxicity, which delays wound healing and weakens the skin, as well as adverse effects such as hand-foot syndrome and peripheral neuropathy, which may impair the ability to perform proper stoma care^17,18^. Additionally, peristomal skin disorders that develop during chemotherapy can substantially impact not only patient QOL but also treatment outcomes for malignant tumors. This effect is attributed to the fact that the presence of skin disorders is sometimes accompanied by interruptions in chemotherapy. Therefore, providing appropriate care may help maintain better clinical outcomes, and preventing the development and progression of peristomal skin disorders is of utmost importance^17^. Additionally, other identified risk factors included a stoma height of less than 10 mm and a loop stoma. Previous reports have already identified low stoma height and loop stoma as risk factors for peristomal skin disorders. This association is likely due to the increased risk of contact between stoma effluent and the surrounding skin, leading to irritant dermatitis^19,20^. In this study, similar findings were observed at later time points during outpatient follow-up, indicating that patients with these risk factors require careful monitoring for the development of peristomal skin disorders over time.

In cases where no peristomal skin disorders were observed at the 1-month outpatient visit, active interventions may not have been routinely performed during subsequent follow-up. However, based on the findings of this study, it is crucial to closely monitor patients with risk factors such as a stoma height of < 10 mm, loop stoma, and those undergoing chemotherapy, because they may be more susceptible to developing peristomal skin disorders. Measures such as more frequent outpatient follow-ups might be necessary for patients with these risk factors.

Notably, no severe peristomal skin disorders were observed in patients who had no peristomal skin disorders at the 1-month outpatient visit in this study. These findings suggest that regularly scheduled outpatient visits might have contributed to early detection and timely management, potentially reducing the progression of severe peristomal skin disorders.

DM was identified as a risk factor for worsening of peristomal skin disorders during outpatient follow-up in patients with peristomal skin disorders at the 1-month outpatient visit. Although continuous intervention likely helps prevent further aggravation, the presence of DM is associated with delayed wound healing and a higher likelihood of worsening skin disorders despite regular outpatient visits^21,22^. This delay is attributed to various factors, including microcirculatory changes and disrupted keratinocyte functions in high-glucose environments^22,23^. Keratinocytes exhibit impaired migration, proliferation, and abnormal expression of matrix metalloproteinases, contributing to poor healing^23^. When evaluation was conducted using the ABCD-Stoma, several risk factors were identified; however, the DET score did not show significant associations with worsening. Although the DET score is reliable for quantitative monitoring, its sensitivity to dynamic or multifactorial changes may be limited because the definition of “severity” is not fully standardized^13,24^. In contrast, monitoring peristomal skin conditions using the ABCD-Stoma may be more appropriate for detecting the progression and severity of peristomal skin disorders.

During outpatient follow-up, severe peristomal skin disorders developed in seven patients (46.7%) among those whose peristomal skin disorders had worsened. The primary risk factor for the development of severe peristomal skin disorders was a stoma height of < 10 mm. Numerous studies have reported that stoma height markedly influences peristomal skin disorders^12,14,25^. As a result, ensuring a stoma height of 10 mm or more is considered essential during stoma creation surgery^14,26^. Furthermore, in this study, the development of severe peristomal skin disorders in patients with a stoma height < 10 mm was observed even during regular outpatient follow-up. Therefore, in cases where the stoma height is less than 10 mm, special attention should be given not only to the development of peristomal skin disorders but also to the potential for severe progression during outpatient follow-up.

This study has some limitations. First, while chemotherapy was identified as a risk factor for peristomal skin disorders during outpatient follow-up, specific treatment regimens and durations were not clarified. Although epidermal growth factor receptor-targeted monoclonal antibodies are known to cause skin disorders, this study alone could not determine which regimens or durations contributed to an increased risk. Second, since the study was conducted in Japan, the findings may not be generalizable to other countries where variations in stoma care practices, healthcare systems, and patient demographics exist. It should also be noted that part of the observation period (2020–2021) overlapped with the coronavirus disease pandemic. Outpatient schedules and patient behavior may have been affected by infection-control restrictions, potentially influencing follow-up frequency and early detection of skin disorders. Third, this prospective multicenter study focused exclusively on malignant rectal tumors, limiting the applicability of its findings to other conditions, such as inflammatory bowel disease or benign stoma-related conditions. In addition, although evaluators were trained and scoring criteria standardized, inter-facility variations in patient management and stoma-care resources could not be completely excluded. Despite these limitations, this study highlights the importance of ongoing support in stoma outpatient care, particularly for patients at higher risk of developing peristomal skin disorders. Targeted monitoring and timely interventions may help identify early signs of deterioration and are associated with better QOL and treatment continuity through continued cancer treatment.

In conclusion, patients with loop stomas, a stoma height of < 10 mm, or those undergoing chemotherapy are at a higher risk of developing peristomal skin disorders during stoma outpatient follow-up. Additionally, DM may increase the likelihood of worsening peristomal skin disorders, while a stoma height of < 10 mm is associated with a greater risk of severe peristomal skin disorders. These findings underscore that continuous monitoring and early intervention in high-risk patients may facilitate prompt detection and management of peristomal skin disorders, thereby maintaining patient outcomes and QOL.

## Data Availability

The datasets used and/or analyzed during the current study available from the corresponding author on reasonable request.
